# Drop jumps versus sled towing and their effects on repeated sprint ability in young basketball players

**DOI:** 10.1186/s13102-021-00395-w

**Published:** 2022-01-04

**Authors:** Alessandro M. Zagatto, Gabriel M. Claus, Yago M. Dutra, Rodrigo A. de Poli, Vithor H. F. Lopes, Stuart Goodall, Irineu Loturco, Daniel Boullosa

**Affiliations:** 1 Post-Graduate Program in Movement Sciences, Laboratory of Exercise Physiology and Sport Performance (LAFIDE), Department of Physical Education, School of Sciences, Sao Paulo State University (UNESP), Av. Eng. Luiz Edmundo Carrijo Coube, 14-01, Vargem Limpa, Bauru, SP CEP 17033-360 Brazil; 2 Department of Sport, Exercise, & Rehabilitation, Faculty of Health of Life Sciences, Northumbria University, Newcastle upon Tyne, UK; 3 Nucleus of High Performance in Sport, São Paulo, Brazil; 4 Federal University of Mato Grosso do Sul, Campo Grande, Brazil

**Keywords:** Neuromuscular fatigue, Physical fitness, Sport, Post-activation potentiation, Post-activation performance enhancement

## Abstract

**Background:**

The aim of the investigation was to compare the occurrence of post-activation performance enhancement (PAPE) after drop jumps, or heavy sled towing, and the subsequent effect on repeated sprint ability (RSA).

**Methods:**

Ten young basketball players (17 ± 1 yrs) performed, in randomized order, RSA test with changes of direction after a standardized warm up followed by drop jumps, heavy sled towing, or no exercise (control condition). Neuromuscular assessments composed of two maximal voluntary contractions of the knee extensors, peripheral nerve stimulation, and surface electromyography (EMG), responses were recorded before and immediately after the RSA. The EMG signal of leg muscles during sprinting were also recorded as well as the blood lactate concentration.

**Results:**

The drop jumps improved the RSA mean time (*P* = 0.033), total time (*P* = 0.031), and slowest time (*P* = 0.029) compared to control condition, while heavy sled towing did not change RSA outcomes (*P* > 0.05). All conditions exhibited a decrease of doublet high frequency stimulation force (pre-post measurement) (*P* = 0.023) and voluntary activation (*P* = 0.041), evidencing the occurrence from peripheral and central components of fatigue after RSA, respectively, but no difference was evident between-conditions. There was a significantly greater EMG activity during sprints for the *biceps femoris* after drop jumps, only when compared to control condition (*P* = 0.013).

**Conclusion:**

Repeated drop jumps were effective to induce PAPE in the form of RSA, while heavy sled towing had no effect on RSA performance in young basketball players. Furthermore, both conditioning activities exhibited similar levels of fatigue following the RSA protocol. Thus, drop jumps may be used as an alternative to induce PAPE and thus improve performance during sprints in young male basketball players.

**Supplementary Information:**

The online version contains supplementary material available at 10.1186/s13102-021-00395-w.

## Background

Basketball is a team sport which consists of brief high-intensity efforts with frequent changes of direction, interspersed with short recovery intervals [1–4]. These high-intensity actions during the match may induce a higher glycolytic pathway contribution with concomitant decrease of high-intensity demands (~ 16% decrement) during the second half of a match [5], which may be associated to a ~ 5% reduction in force production of the knee extensors [6]. Therefore, identification of effective strategies to increase high-intensity actions, whilst minimizing the effects of fatigue during matches, is an important objective for coaches and practitioners. Specifically, in basketball, competitive players have frequency of occurrences during an official match of 1.06 ± 0.52 sprints and 1.13 ± 0.42 jumps per minute [3], evidencing the relevance of both attributes to basketball performance. Furthermore, recent evidence suggests that repeated sprints with directional changes are more related to playing demands of basketball matches than single sprint efforts, or sprints performed in a linear fashion [2, 7]. Therefore, the use of ergogenic sources that can enhance performance during these specific actions should be a priority for elite basketball players and researchers in the area.

Post-activation potentiation (PAP) is a potential strategy to improve performance during high-intensity activities in basketball. PAP is defined as an acute involuntary improvement in muscle force generating capacity (generally confirmed via an evoked stimulation) [8], after a maximal, or submaximal, conditioning activity (CA) [9]. More recently, the term post-activation performance enhancemnet (PAPE) has been introduced in the literature to differentiate between potentiation responses observed with twitch verification (i.e. PAP) or during athletic tasks, mostly explosive actions such as jumps and sprints (i.e. PAPE). While some mechanistic differences between PAP and PAPE have been proposed [10], there is no current consensus on the issue [11]. In fact, PAPE and PAP responses have been simultaneoulsy reported following an array of exercise protocols [8, 12, 13], however, PAPE can arise without ocurrance of PAP. For example, previous studies have reported performance improvements during high-intensity efforts (i.e., jumps and sprints) after very different CAs comprising heavy-load and/or ballistic exercises [14–16]. In this context, there is recent evidence suggesting the effectiveness of sled towing on acceleration performance in recreationally trained adult males [17] and high school football players [18]. Meanwhile, it was revealed that drop jumps may be effective in improving repeated sprint ability (RSA) with changes of direction in adult basketball players [1]. However, it is unknown if heavy sled towing would be as effective as drop jumps, for enhancing peformance of repeated high-intensity efforts. Considering the potential influence of age on PAPE/fatigue responses [19], it is pertinent to compare the effects of these different CAs in a group of young basketball players. The current knowledge on PAPE strategies for young athletes is limited and, to the best of our knowledge, there is no information regarding young basketball players. This information would be also important to better select conditioning exercises which are commonly combined during complex training sessions in team-sport disciplines [20].

Accordingly, the purpose of this study was to investigate and compare the use of drop jumps and heavy sled tows as CAs to improve performance during repeated sprint efforts, with directional changes. Additionally, we examined the effects of these protocols on selected markers of fatigue. Our hypotheses were that both, drop jumps and heavy sled towing, would induce PAPE, however, the drops jumps would be more effective due to lower level of residual fatigue.

## Methods

### Participants

The minimal required sample size was determined using G*power software [21]. The input parameters used for *F* test family were alpha = 0.05 and power = 0.90. Using the time until exhaustion during cycling at a supramaximal power output after control (157 ± 42 s) and the one preceded by drop jumps protocol (171 ± 44 s; de Poli et al. 2020), the minimal required sample size of 9 participants was estimated. A basketball team was contacted and fifteen young male basketball players (the players were from the under 17 (n = 7) and 19 (n = 8) categories and had experience of competing at a national level) were recruited to participate in the current study after meeting the following inclusion criteria: (1) basketball training experience ≥ 4 years; (2) absence of recent (< 3 months) musculoskeletal and joint injuries; and (3) absence of recent (< 3 months) regular use of any ergogenic substance (e.g., creatine, beta-alanine). The exclusion criteria were: (1) suffering an injury during the period of investigation (n = 2); (2) not tolerate the assessment procedures (n = 1); and (3) withdraw from the team (n = 1). Finally, 10 participants (age: 17.5 ± 1.2 years; stature: 1.91 ± 0.07 m; body mass: 87.2 ± 15.4 kg; competitive experience: 5.2 ± 1.5 years) were selected. Prior to the commencement of the study, the athletes were informed about benefits and risks of experimental procedures and signed an informed consent form. For the underage participants, their parents were informed and signed the consent form. All experimental procedures of the present study were approved by the Research Ethics Committee of the Sao Paulo State University (#2.540.512/2018) according to the Declaration of Helsinki.

### Experimental design

A randomized cross-over design was adopted and all sessions were completed at the end of the pre-season and at the beginning of the competitive season, over a 3-week period. All assessments were performed on the same official basketball court, at the same time of day, with a consistent temperature of 29.3 ± 2.8 °C and a relative humidity of 52.2 ± 8.5%. All exercise sessions were separated with a minimum of 48 h and a maximum of 72 h.

Firstly, participants completed a familiarization session to the RSA test, to the CAs (i.e., drop jump and resisted sled towing), and to the neuromuscular assessments. In the following two sessions, participants initially performed a standardized warm-up (2 min of submaximal jogging, 1 min of side-to-side submaximal running, and 2 min of intermittent 10 m submaximal high-intensity run). After 5 min of passive rest, a protocol for the evaluation of neuromuscular function (i.e., measurement of central and peripheral fatigue, see below) was conducted. Following neuromuscular evaluation, participants performed one of the CAs (one trial for each CA) or remained at rest for 5 min (i.e., control condition). After interventions (4 min for drop jumps [considering the time spent during CA resulting in an interval ~ 5-min] and 8 min for heavy sled towing [considering the time spent during CA resulting in an interval ~ 8.2- min]), the RSA test was conducted with simultaneous recording of surface electromyography (EMG) of lower limb muscles. The reason for different intervals after CAs is reported in “Conditioning Activities” section below. Immediately after RSA testing, neuromuscular function was assessed. Finally, capillary blood samples were collected before each RSA test and at 3, 5, and 7 min post, to determine blood lactate concentration.

### Repeated sprint ability test (RSA)

The RSA test, which included a change of direction (“L” format), used in this study consisted of 10 × 30 m maximal sprints with each sprint involving 5 rapid directional changes (6 runs of 5 m), interspersed with 30 s of passive recovery. This test was selected because it has been previously shown to mimic real game-play demands, with good reliability [2, 22]. All sprints were recorded by a 30 Hz digital camera (GoPro Hero 3 + Black, San Mateo, CA, USA) that recorded the start and the end of sprinting bouts. Subsequently, the recordings were analyzed using a custom-made software (v.0.8.15, Kinovea, Open source for Windows) to determine the sprinting time. The time to complete each sprint was used to determine the best time, mean time, slowest time, and total time (i.e., the accumulate performance time of 10 sprints only) of the RSA test.

### Neuromuscular assessment

#### Force data acquisition

Participants performed two 5 s maximal voluntary contractions (MVCs) with the knee extensors of their self-reported dominant leg, interspersed with 1 min of passive rest [8, 23, 24]. The two MVCs were performed at baseline and as soon as possible after (77.50 ± 21.49 s) the RSA test. The measurements were carried out in a specific chair designed for maintaining hip and knee flexion at 90°, with participants secured by straps [8, 23, 24]. A metal rod was attached to the ankle and connected to a load cell with a maximum capacity of 100 kgf (MK Controle, São Paulo, SP, Brazil). The load cell signal was acquired by an analog data acquisition mode (NI 6009, National Instruments, Austin, TX, USA) sampling at 1000 Hz, using Labview software (National Instruments, Signal express, Austin, TX, USA). The recorded data were subsequently filtered by a second-order Butterworth filter and analyzed with custom designed software (MatLab R2015b, MathWorks, Natick, MA, USA). Before each test, the load cell was calibrated using known weights to create a linear regression model (r^2^ > 0.99). The peak force of MVCs was defined as the mean force recorded during 100 ms of the force plateau [8, 24].

### Peripheral nerve stimulation

Peripheral nerve stimulation was delivered over the femoral triangle (cathode), and anterior to the greater trochanter of the femur (anode) with a high-voltage electric stimulator (Bioestimulador V2 400 V peak to peak, Insight, Brazil). A Ag/AgCl electrode (recording area, 78.5 mm^2^; Medi-trace, Dublin, Ireland) was used for cathode and a 5 × 5 cm electrode (Valutrode, CF5050 model, Brazil) used for anode [24]. The optimal intensity of stimulations was determined individually before each session by the application of consecutive incremental doublets to the relaxed muscle (square-wave, 100 Hz, pulse duration of 1 ms, initial intensity of 80 mA with 20 mA increments) until a plateau in force was reached [8, 24]. The maximal electrical current achieved (mA) was recorded, and supramaximal stimulation was ensured by increasing the final intensity by ~ 20% (253 ± 58 mA) [23, 24].

Doublet, high-frequency stimulation (i.e., square-wave, 100 Hz, pulse duration of 1 ms) were delivered during MVCs (~ 2 s), followed doublets at high frequency (Db100; stimulation frequency in Hertz) 5 s after MVCs, a single stimulus was delivered at 10 s and finally a low frequency stimulation (Db10; stimulation frequency in Hertz) was delivered at 15 s after MVC, with the muscle in a relaxed state [25]. The amplitude of the force signal superimposed by the doublet high frequency stimuli during MVCs, and the force produced by the high frequency stimuli at rest after MVCs were used to measure the percentage of voluntary activation (VA) through Eq. 1 [23, 24, 26]. The ratio between the force amplitudes produced by the doublets of low and high frequencies at rest, were subsequently calculated and used as an index of low-frequency muscle fiber depression [26].

1 $$\% {\text{VA}} = \left\{ 1 - [{\text{Superimposed}}\;{\text{force}} \times \left( {\frac{{{\text{Force}}\;{\text{level}}\;{\text{at}}\;{\text{stimulation}}}}{{{\text{Peak}}\;{\text{force}}}}} \right)/{\text{high}}\;{\text{frequency}}\;{\text{force}}]\right\} \times 100$$

### Surface electromyography

A wireless EMG device (Cometa System, Italy) was used during the entire RSA test, while a fixed cable EMG device (Miotec, Brazil) was used during the neuromuscular assessments [24], with both devices sampling at 2000 Hz. The Ag/AgCl electrodes (recording area, 78.5 mm^2^; Medi-trace, Dublin, Ireland) were placed over the prepared skin (shaved skin and gently cleaned by abrasion with fine sandpaper and alcohol 70%) of the *vastus lateralis* (1/3 distal) to measure EMG responses during MVCs, with a ground electrode placed on the ulnar styloid process. During the RSA test, further electrodes were placed on the *gastrocnemius medialis, rectus femoris, vastus lateralis,* and *biceps femoris* as previously described [24].

The EMG signal was subsequently band-pass filtered (20–500 Hz). During the assessment of neuromuscular function, the root mean square (RMS) and median frequency (MdF) of 1 s of force plateau were used as indices of general neuromuscular discharge magnitude and rate, respectively [24]. The peak-to-peak maximal amplitude evoked by single stimulus (M-wave and [M-wave_amp_]) were calculated and used as indices of sarcolemmal excitability [24]. During the RSA test, all values of RMS and MdF were normalized by the first sprint value of the control condition. The RMS and median frequency were calculated using the LabChart Pro v.8 Software (ADInstruments, Colorado Springs, CO, USA).

### Conditioning activities

#### Identification of optimal recovery interval for conditioning protocols

Prior to experimental sessions, pilot work with 6 physically-active males (age: 24.0 ± 2.5 years) was conducted to determine the optimal recovery interval (4 vs. 8 min) after both CAs, before completing the RSA test. For drop jumps, all the RSA test outcomes measured after 4 min of recovery were statistically better compared to control condition (RSA test performed without previous CA) (*P* = 0.048 for best time; *P* = 0.020 for mean time; *P* = 0.020 for total time, and *P* = 0.013 for slowest time) than those measured at 8 min of recovery (*P* = 0.508 for best time; *P* = 0.102 for mean time; *P* = 0.087 for total time, and *P* = 0.093 for slowest time) (see Additional file 1: Table S1), while for the heavy sled towing, only the mean time and total time were significantly lower compared to control condition after 8 min of recovery (*P* = 0.034 and *P* = 0.029, respectively) (see Additional file 1: Table S2). Therefore, the RSA test was performed 4-min after drop jumps, and 8 min after the heavy sled towing. All CAs were realized on the same court that RSA was performed.

### Drop jumps

The drop-jump protocol consisted of 1 set of 5 repetitions, interspersed with 15 s of passive recovery [8, 14]. The drop-jump height was individually determined during the familiarization sessions. All participants performed 1 set with 3 jumps from four different heights (40, 60, 80, and 100 cm) on a force plate (Cefise, Nova Odessa, Brazil) and the box height selected was that exhibiting the highest reactive strength index (RSI; i.e., the ratio of jump height to ground contact time) [27].

### Heavy sled towing

The heavy sled towing protocol composed of 1 sprint of 15 m with a sled attached to the waist, which was loaded with 75% of the players’ body mass [16].

### Blood sample collection and analysis

Blood samples (25 µL) were collected at rest and 3 min after the RSA test from the earlobe, using heparinized capillary tubes. Blood samples were immediately deposited into microtubes containing 50 µL of sodium fluoride at 1%, and frozen at − 20 °C for posterior analyses (YSI 2900, YSI, Yellow Springs, Ohio, EUA).

### Statistical analyses

The results are presented as means ± standard deviations and 95% confidence intervals (CI95%). A two-way repeated measures analysis of variance (ANOVA) was used to identify the effect of time and condition on neuromuscular assessment parameters. A repeated measures of ANOVA was used to compare RSA test performance outcomes and the percent changes of neuromuscular assessments between conditions. In all cases, the Mauchly´s test of sphericity was applied, and the Greenhouse–Geisser Epsilon correction was used when the sphericity criteria were not met. When necessary, the analyses were completed with SIDAK post hoc test. A significance level of *P* ≤ 0.05 was assumed in all cases. All statistical analyses were performed using the software SPSS version 20 (IBM Corp., Chicago, IL, USA). *Cohen’s d* effect size [ES(CI95%)] for the RSA outcomes and the delta changes between conditions for parameters of neuromuscular assessment was also calculated.

## Results

**Fig. 1:**
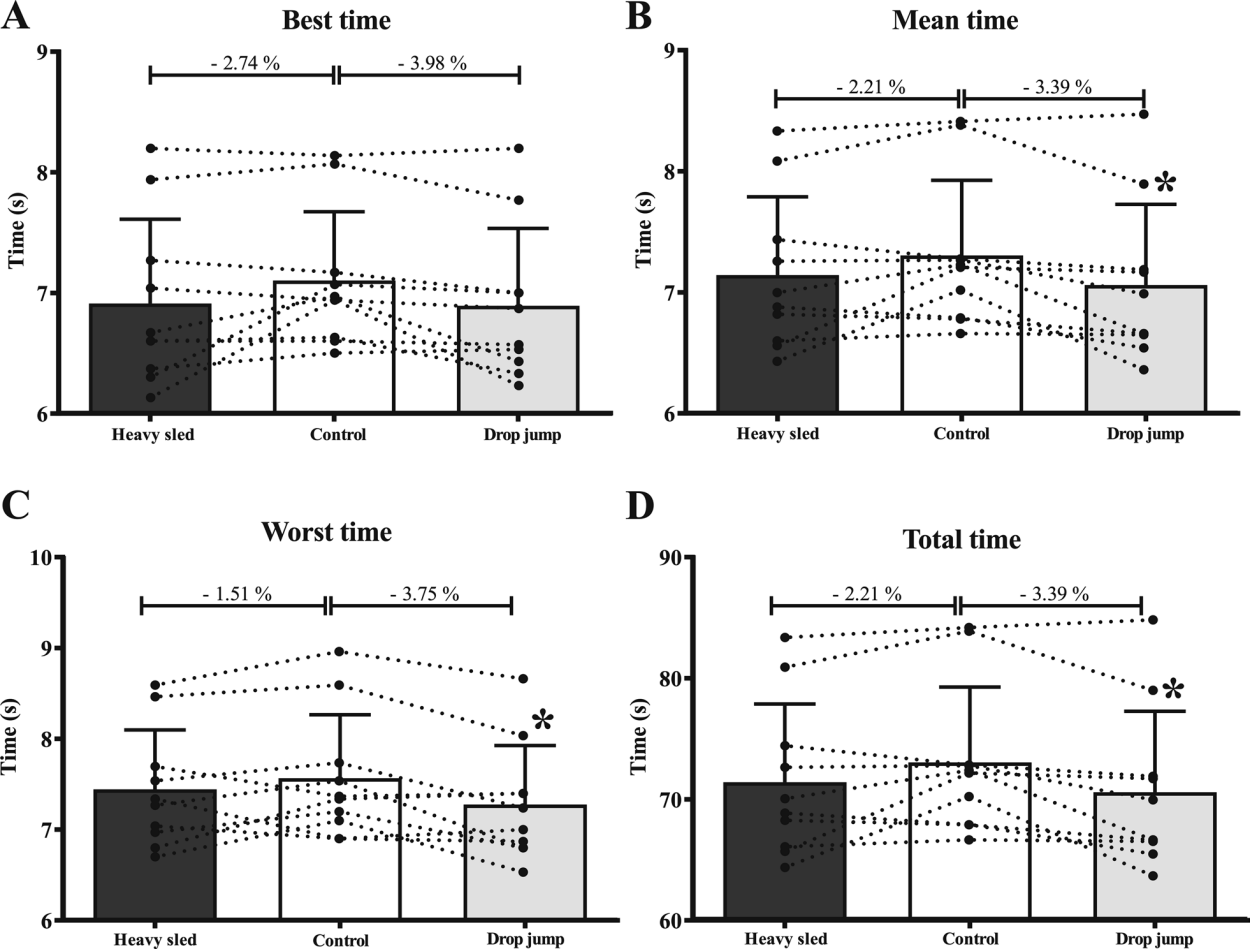
RSA performance outcomes.: Best time performance (**A**); Mean time performance (**B**); Worst time performance (**C**); and Total time performance (**D**). Data are presented mean ± SD, and the percentage difference between the conditions of conditioning activity with the control condition n = 10). * = different from control condition (*P* < 0.05)

The results of RSA performance during control, drop jumps and heavy sled towing conditions are presented in Fig. 1. Significant differences were found to mean time (ANOVA *p*-value = 0.033), slowest time (ANOVA *p*-value = 0.029), total time (ANOVA *p*-value = 0.031), while best time (ANOVA *p*-value = 0.073) did not change. RSA outcomes were different between the drop jump and control condition with improvements shown following drop jumps in mean time [mean Δ = − 0.18 s; *P* = 0.039; ES = − 0.35 (CI95%:− 0.60 to − 0.09)], total time [mean Δ = − 1.85 s; *P* = 0.037; ES = − 0.35 (CI95%: − 0.60 to − 0.10)]and slowest time [mean Δ = − 0.22 s; *P* = 0.045; ES = − 0.41 (CI95%: − 0.71 to − 0.10)]. However, heavy sled towing did not present any differences compared to the drop jumps (ESs = 0.02 (CI95%: − 0.25 to 0.30) for best time, 0.12 (CI95%: − 0.18 to 0.41) for mean time, 0.12 (CI95%: − 0.17 to 0.41)for total time, and 0.23 (CI95%: − 0.08 to 0.55)for slowest time and control conditions (ESs(CI95%) = − 0.25 (CI95%: − 0.57 to 0.07), − 0.23 (CI95%: − 0.51 to 0.05), − 0.23 (CI95%: − 0.51 to 0.05), and − 0.17 (CI95%: − 0.49 to 0.15), respectively (*P* ≥ 0.110).

**Fig. 2:**
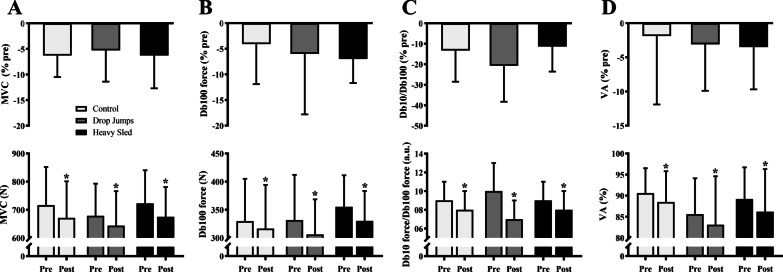
Neuromuscular assessments measured before and after the RSA.: MVC peak force (**A**), Db100 force (**B**), Db10/Db100 ratio (**C**) and voluntary activation (VA; **D**) (n = 10). Values are expressed as Mean ± SD and the percentage difference between pre and post values. * = *P* < 0.05 after vs. before RSA testing in the same condition

Neuromuscular evaluation outcomes before and after RSA testing for all conditions are presented in Table 1 and in Fig. 2. There was no interaction between conditions for peak MVC peak force (*P* = 0.821), RMS (*P* = 0.684) and MdF (*P* = 0.904). An effect for time was found for MVC force (mean Δ =  − 6.0%; *P* = 0.001). In addition, a main effect for MdF during MVCs revealed a higher value after RSA for all conditions (mean Δ = 9.3%; *P* = 0.023). There was no interaction between conditions for force at Db100 (*P* = 0.591), the ratio Db10/Db100 (*P* = 0.126), as well as for M-wave_amp_ (*P* = 0.509). A main effect for time existed with a decrease after RSA for force at Db100 (mean Δ =  − 6.3%; *P* = 0.023) and the ratio Db10/Db100 (mean Δ =  − 15.6%; *P* = 0.003). Finally, there was no interaction between conditions for VA (*P* = 0.953), however, a main effect of time revealed the existence of central fatigue in all experimental conditions (mean Δ =  − 2.9%; *P* = 0.041). The delta changes between conditions for all parameters of neuromuscular assessment did not differ (*P* > 0.05) (Table 1 and Fig. 2) and the effects sizes were of trivial to small (Table 2).

**Table 1:** Neuromuscular assessment

	Control			Heavy sled			Drop jumps		
	Before	After	Δ%	Before	After	Δ%	Before	After	Δ%
*Global function indicators*									
*Vastus lateralis* RMS (*µ*V)	347.90 ± 128.20 (256.19 to 439.65)	334.30 ± 138.30 (235.38 to 433.22)	− 3.90 ± 18.00 (− 16.82 to 8.98)	362.20 ± 183.90 (231.68 to 494.76)	319.30 ± 132.60| (224.44 to 414.21)	− 8.70 ± 20.00 (− 23.07 to 5.58)	315.50 ± 114.10 (233.87 to 397.05)	304.80 ± 133.70 (109.10 to 400.44)	− 3.70 ± 23.70 (− 20.67 to 13.22)
*Vastus lateralis* MdF (Hz)	70.80 ± 13.30 (61.32 to 80.32)	75.80 ± 8.00* (70.10 to 81.57)	10.30 ± 23.70 (− 6.66 to 27.25)	65.40 ± 14.10 (55.28 to 75.44)	73.40 ± 14.80* (62.78 to 83.92)	14.00 ± 21.60 (− 1.44 to 29.42)	66.20 ± 11.70 (57.86 to 74.58)	72.00 ± 12.60* (63.02 to 81.02)	13.10 ± 32.70 (− 10.29 to 36.50)
*Peripheral function indicators*									
M-wave_amp_ (mV)	12.10 ± 3.30 (9.81 to 14.47)	12.80 ± 3.30 (10.42 to 15.14)	6.40 ± 13.70 (− 3.32 to 16.26)	12.60 ± 3.10 (10.41 to 14.85)	13.50 ± 4.60 (10.24 to 16.87)	6.00 ± 16.10 (− 5.47 to 17.55)	12.10 ± 3.70 (9.44 to 14.74)	12.30 ± 4.30 (9.17 to 15.39)	1.20 ± 12.60 (− 7.81 to 10.29)

Values are expressed as Mean ± SD (CI95%). * = *P* < 0.05 after vs. before RSA testing in the same condition. RMS = root mean square; MdF = Median frequency; M-waveamp = maximal amplitude of m-wave

**Table 2:** Effect Sizes (ES) of delta changes between conditions for parameters of neuromuscular assessment

	Heavy Sled—Control	Drop Jumps—Heavy Sled	Drop Jumps—Control
**Global function indicators**			
MVC force	0.00 (− 1.49 to 1.49)	0.22 (− 1.40 to 1.84)	0.22 (− 0.77 to 1.20)
*Vastus lateralis* RMS	− 0.24 (− 1.14 to 0.80)	0.25 (− 0.59 to 1.10)	0.01 (− 1.18 to 1.20)
*Vastus lateralis* MdF	0.14 (− 0.87 to 1.16)	− 0.03 (− 0.98 to 0.91)	0.11 (− 1.21 to 1.43)
**Peripheral function indicators**			
M-wave_amp_	− 0.03 (− 0.82 to 0.77)	0.32 (− 1.09 to 0.46)	− 0.35 (− 1.12 to 0.43)
Db100 force	− 0.34 (− 1.13 to 0.45)	0.11 (− 0.83 to 1.06)	− 0.23 (− 1.25 to 0.79)
Db10/dB100	0.11 (− 0.46 to 0.68)	− 0.56 (− 1.32 to 0.21)	− 0.45 (− 1.07 to 0.17)
**Central Fatigue indicators**			
VA	− 0.15 (− 0.98 to 0.68)	0.04 (− 0.51 to 0.58)	− 0.11 (− 1.00 to 0.78)

Values are expressed as Effect Size (CI95%). MVC = voluntary maximal contraction; RMS = root mean square; MdF = Median frequency; M-waveamp = maximal amplitude of m-wave; Db100 = doublets at high frequency; Db10 = low frequency stimulation; VA = voluntary activation

**Fig. 3:**
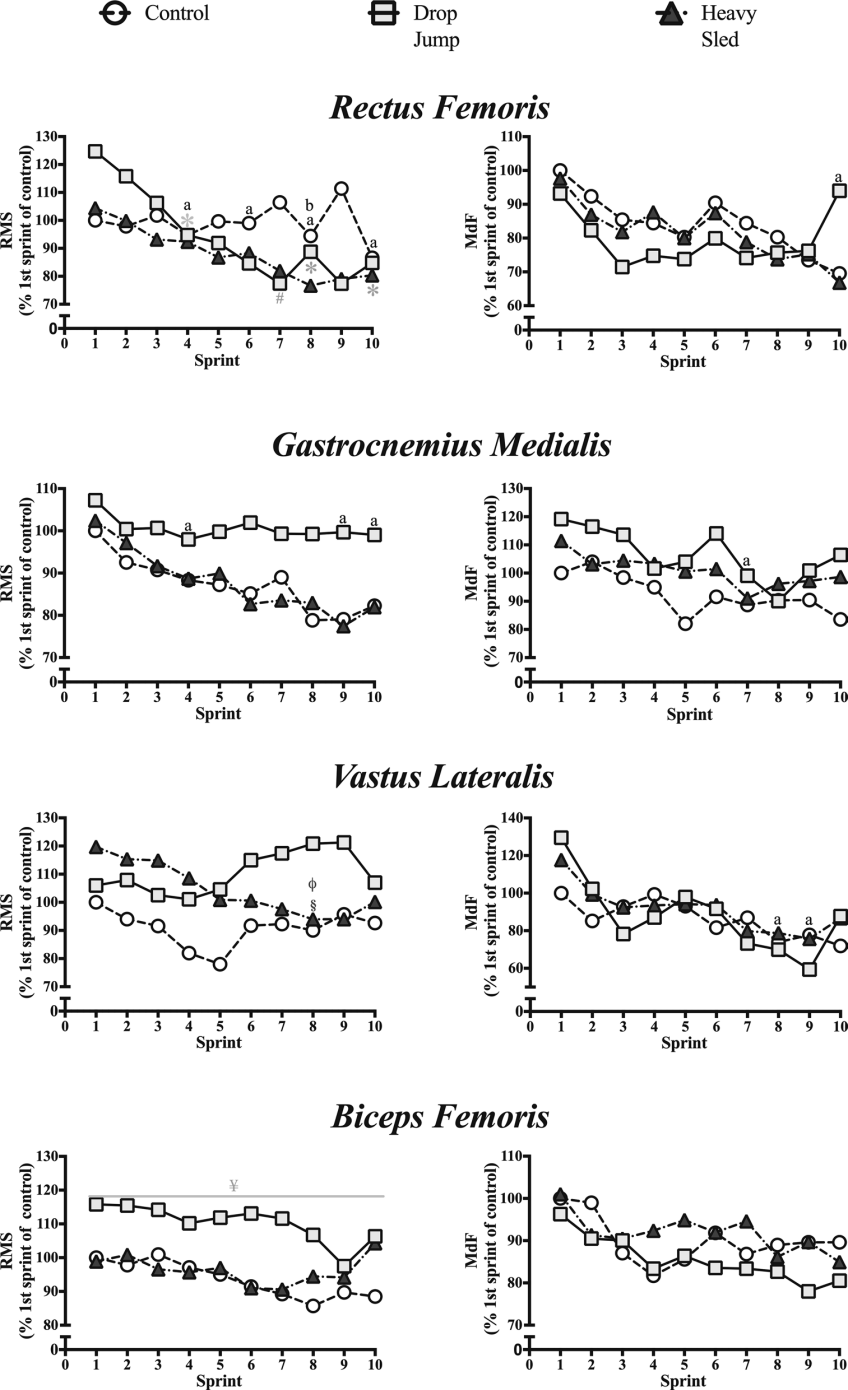
EMG activity of selected lower-limb muscles measured during repeat-sprint ability test, normalized by the first sprint values observed in control condition (n = 10). a = main time effect, significant difference from 1^st^ sprint; b = main time effect, significant difference from 2^nd^ sprint; ¥ = main condition effect, significant difference from control condition; * = interaction effect, significant difference from 1^st^ sprint in drop jump condition; # = interaction effect, significant difference from 2^nd^ sprint in drop jump condition § = interaction effect, significant difference from 1^st^ sprint in heavy sled condition; φ = interaction effect, significant difference from 2^nd^ sprint in heavy sled condition

The EMG data during RSA testing is presented in Fig. 3. The most relevant findings were a reduction in RMS of the *rectus femoris* in the fourth sprint, in the eighth sprint, and in the tenth sprint when compared to the first sprint, in the drop jump condition (mean Δ =  − 24.9%; *P* = 0.021, mean Δ =  − 28.7%; *P* = 0.08, and mean Δ =  − 30.7%; *P* = 0.035, respectively). There was also a difference between conditions for RMS in the *biceps femoris,* showing higher RMS values in the drop jumps condition, compared to control (mean Δ = 16.4%; *P* = 0.013).

No difference between conditions was observed for lactate concentrations at baseline (control: 0.77 ± 0.32 mmol L^−1^; heavy sled towing: 0.83 ± 0.29 mmol^.^L^−1^; drop jumps: 0.93 ± 0.57 mmol L^−1^; *P* = 0.655) and after the RSA (control: 5.93 ± 1.67 mmol L^−1^; heavy sled towing: 6.55 ± 1.88 mmol L^−1^; drop jumps: 6.27 ± 0.68 mmol L^−1^; *P* = 0.617). Besides, the drop-jump condition presented a lower significant difference for lactate concentrations prior to RSA testing when compared with other conditions (control: 1.91 ± 0.75 mmol L^−1^; heavy sled towing: 1.81 ± 0.25 mmol L^−1^; drop jumps: 1.12 ± 0.44 mmol L^−1^; *P* = 0.002; post hoc control vs. drop jumps: *P* = 0.015; post hoc heavy sled towing vs. drop jumps: *P* = 0.003).

## Discussion

The purpose of the present study was to investigate the effectiveness of drop jumps and heavy sled towing as conditioning activities to enhance the performance of a specific basketball repeated sprint test. Our findings suggest that a drop-jump protocol can elicit a performance enhancing effect on RSA relative to the control condition in young basketball players, while heavy sled towing was not effective. Furthermore, fatigue was evident in all experimental conditions, suggest the occurrence of a peripheral mechanism (i.e. PAP) in the PAPE response. Specifically, the drop-jump condition exhibited a higher RMS in the *biceps femoris* only during the RSA testing.

The improvement in RSA performance after drop jumps may be important for basketball performance with effect size varying among small to moderate. A previous study has reported a correlation between RSA mean time, and distance covered during high intensity actions throughout matches [28]. In a recent study, Zagatto et al. [1] investigated the PAPE effects of drop jumps (1 × 5 drop jumps before a first RSA testing, and 1 × 3 drop jumps before a second RSA) in professional basketball players, and also found a greater best time, total time, and mean time in both RSA tests after the drop jumps condition, thus corroborating the current findings. In the present study, a greater mean time, total time, and reduction on the slowest time after drop jumps was found. Furthermore, these results may be associated with the specific demands of basketball since the RSA tests were performed with successive directional changes [2, 29].

The improvement in RSA performance induced by drop jumps may be attributed to a potential PAP effect; however, for methodological reasons, we did not measure this occurrence with the twitch verification in the present study. Previously, de Poli et al. [8] revealed that 1 set of 5 repetitions interspersed with 15 s of recovery induced PAP improving the force evoked by a high frequency stimulus (i.e. Db100 Hz) thus confirming the effectiveness of drop jumps to evoke PAP. In the current study we did not perform neuromuscular evaluations immediately following the conditioning activities to confirm PAP, to avoid possible accumulated twitch potentiation due to repeated MVCs and electrical stimulations. Concerning the absence of beneficial effect for the heavy sled towing condition, we speculate that it could be attributed to the biomechanical characteristics of this exercise. For instance, while the usual duration of the drop jumps is < 300 ms, heavy sled towing led to much longer sprint times when compared to the unloaded sprints. Although not measured prior to the RSA, the heavy sled towing could have elicited muscle fatigue, similar to when heavy resistance exercise is used as a conditioning activity [30], and that could help to explain no improvement in performance. The effectiveness of drop jumps in comparison to heavy sled towing, supports the results of meta-analysis by Seitz and Haff [15] that presented greater effect sizes for ballistic exercises (0.47) when compared to high-intensity (0.41), moderate intensity, (0.19) and isometric (− 0.09) exercises.

The main results for RMS during the RSA test showed a significant reduction in RMS of *rectus femoris* from the fourth, eighth, and tenth sprints in the drop-jump condition, when compared to the first sprint, and a higher level of activity in the drop-jump condition compared to the control. The RMS of the EMG is considered as a global index of motor unit recruitment for the specific exercise performed. One of the proposed mechanisms for PAP is the enhanced recruitment of higher order motor units following a CA [9]. Thus, the absence of changes or the reduction in RMS during RSA sprints in the agonist muscles suggests the progressive development of fatigue and that this mechanism may be not considered after heavy sled towing and drop jumps. Previously, Aandahl et al. [31] found an increase of the kick velocities (+ 3.3%) and the RMS of the *rectus femoris* after a taekwondo kick movement with an elastic band. More recently, de Poli et al. [8] investigated the effect of drop jumps on supramaximal cycling until exhaustion and reported the increment in performance until exhaustion (+ 9.8%) without interaction between drop-jump and control conditions for RMS of agonist muscles (i.e. *rectus femoris*, *vastus lateralis*, *vastus medialis* and *gluteus maximus*). These recent results are in agreement with another recent study by Zagatto et al. [1], who measured the EMG signal during RSA sprints after drop jumps in professional basketball players but without changes in EMG within the muscles evaluated. However, the greater RMS found for the *biceps femoris* in the drop-jump condition, may partially explain the PAPE observed as it would be related to a lower fatigue of this muscle when compared to the other conditions [32]. This, in turn, would favor a greater stiffness during sprinting bouts and thus, a better performance, not only during linear sprinting but also during successive directional changes [33]. However, the fact that we averaged RMS values during all the sprinting bouts, without differentiating between stride or acceleration phases, limits our understanding. Therefore, further studies should precisely elucidate the role of this acute adaptation during sprinting after performing drop jumps as a CA and consider the changes at a motor unit level.

It is interesting to note that despite sprinting performance was greatest following use of drop jumps protocol, the assessment of neuromuscular function revealed similar fatigue levels post-exercise in all trials. The results showed a combination of a decrement in peripheral (Db100 and ratio Db100/Db10) and central parameters (%VA and RMS/M-wave_amp_) induced by repeated sprinting, corroborating previous work [23, 26, 34, 35]

The main limitation of the current study was the absence of twitch verification to confirm the existence of PAP or fatigue prior to the RSA. However, de Poli et al. [8] recently showed, through a similar experimental approach, that drop jumps are able to evoke PAP, thus confirming the influence of PAP on PAPE.

## Perspective

The current results confirm the validity and efficiency of a “short” drop-jump protocol (i.e., 1 set of 5 repetitions) to improve the linear and multilinear sprint performance of young basketball players. As this exercise is very easy to prescribe and implement, it may be regularly included at the end of warm-up and/or re-warm up sessions during basketball games.

## Conclusion

Drop jumps were effective to induce PAPE via improvements in repeated sprinting ability, while heavy sled towing elicited no effect in young basketball players. Furthermore, both activities induced similar levels of fatigue after the repeated sprinting protocol.

## Supplementary Information

**Additional file 1.** **Supplementary Material: Supplementary Table 1.** Outcomes of optimal recovery interval related to conditioning activity drop jumps. **Supplementary Table 2.** Outcomes of optimal recovery interval related to conditioning activity heavy sled towing.

## Data Availability

Can be made available upon reasonable request.

## Abbreviations

PAPE: Post-activation performance enhancement
RSA: Repeated sprint ability
EMG: Surface electromyography
PAP: Post-activation potentiation
CA: Conditioning activity
Db100: Doublets at high frequency
Db10: Doublets at low frequency
MVC: Maximal voluntary contraction
VA: Voluntary central neuromuscular activation
RMS: Root mean square
MdF: Median frequency
M-wave_amp_: Amplitude of m-wave
RSI: Reactive strength index
ANOVA: Two-way repeated measures analysis of variance
CI95%: 95% Confidence intervals
