# Supplementary material for: Drop jumps versus sled towing and their effects on repeated sprint ability in young basketball players
Source: BMC Sports Sci Med Rehabil. 2022 Jan 4;14:4. doi: 10.1186/s13102-021-00395-w (PMC8729080; doi:10.1186/s13102-021-00395-w)
Supplement: Supplementary file 1 — Additional file 1. Supplementary Material: Supplementary Table 1. Outcomes of optimal recovery interval related to conditioning activity drop jumps. Supplementary Table 2. Outcomes of optimal recovery interval related to conditioning activity heavy sled towing. [file 13102_2021_395_MOESM1_ESM.docx]

**SUPPLEMENTARY MATERIAL**

**Supplementary Table 1.** Outcome of optimal recovery interval related to conditioning activity drop jumps.

|  | **Control condition** | **4 min** | **8 min** |
| --- | --- | --- | --- |
| **Best time (s)** | 8.03 ± 0.59 (7.30 – 8.77) | 7.92 ± 0.59* (7.20 – 8.65) | 7.95 ± 0.44 (7.41 – 8.49) |
| **Mean time (s)** | 8.34 ± 0.63 (7.57 – 9.11) | 8.13 ± 0.56* (7.43 – 8.82) | 8.17 ± 0.47 (7.59 – 8.75) |
| **Total time (s)** | 83.42 ± 6.25 (75.66 – 91.19) | 81.27 ± 5.57* (74.35 – 88.19) | 81.51 ± 4.67 (75.71 – 87.30) |
| **Worst time (s)** | 8.64 ± 0.61 (7.88 – 9.40) | 8.34 ± 0.57* (7.63 – 9.04) | 8.42 ± 0.49 (7.81 – 9.03) |

Results presented mean ± standard deviation (CI95%). * = different from control condition (*P* < 0.05).

**Supplementary Table 2.** Outcome of optimal recovery interval related to conditioning activity heavy sled towing.

|  | **Control condition** | **4 min** | **8 min** |
| --- | --- | --- | --- |
| **Best time (s)** | 8.28 ± 0.45 (7.63 – 8.88) | 8.15 ± 0.56 (7.41 – 8.96) | 7.95 ± 0.52 (7.30 – 8.60) |
| **Mean time (s)** | 8.54 ± 0.51 (7.83 – 9.25) | 8.42 ± 0.53 (7.67 – 9.12) | 8.20 ± 0.54* (7.54 – 8.86) |
| **Total time (s)** | 85.45 ± 5.13 (78.28 – 92.51) | 84.15 ± 5.29 (76.61 – 91.19) | 82.00 ± 5.30* (75.43 – 88.58) |
| **Worst time (s)** | 8.88 ± 0.79 (7.83 – 10,00) | 8.66 ± 0.57 (7.83 – 9.36) | 8.46 ± 0.56 (7.76 – 9.15) |

Results presented mean ± standard deviation (CI95%). * = different from control condition (*P* < 0.05).
